# Dedifferentiation of Human Cardiac Myofibroblasts Is Independent of Activation of COX-2/PGE_2_ Pathway

**DOI:** 10.3390/ijms23063023

**Published:** 2022-03-11

**Authors:** Vy Tran Luu, Sang Phan, Zhu-Qiu Jin

**Affiliations:** Department of Pharmaceutical and Biomedical Sciences, College of Pharmacy, California Northstate University, Elk Grove, CA 95757, USA; vy.tranluu4045@cnsu.edu (V.T.L.); sang.phan5876@cnsu.edu (S.P.)

**Keywords:** COX-2, dedifferentiation, myofibroblast, PGE_2_, Phorbol 12-myristate 13-acetate

## Abstract

The differentiation of cardiac fibroblasts to myofibroblasts is considered to be a critical step in activation and progression of cardiac fibrosis in heart disease. TGF-β is one of the key cytokines that promotes transition of fibroblasts to myofibroblasts. Dedifferentiation of formed myofibroblasts or reversal of formed myofibroblasts to fibroblasts remains incompletely understood. Prostaglandin E_2_ (PGE_2_) has been shown to dedifferentiate human lung myofibroblasts. The role of activation of the COX-2/PGE_2_ pathway in dedifferentiation of cardiac myofibroblasts remains unknown. Here, we show that phorbol 12-myristate 13-acetate (PMA) but not PGE_2_ induces dedifferentiation of de novo adult human cardiac myofibroblasts stimulated by TGF-β1 from human cardiac fibroblasts as evidenced by reduced expression of α-smooth muscle actin (α-SMA). PMA remarkably increased endogenous levels of PGE_2_ in human cardiac myofibroblasts. Pretreatment of myofibroblasts with NS-398, a selective COX-2 inhibitor, and PF-04418948, a selective PGE_2_ receptor type 2 (EP2) antagonist, had no effect on expression of α-SMA nor abolished the dedifferentiation induced by PMA. Our results indicated that endogenous and exogenous PGE_2_ has no effects on dedifferentiation of cardiac myofibroblasts. PMA-induced dedifferentiation of cardiac myofibroblast is independent of activation of COX-2 and PGE_2_ pathway. The mechanism in PMA-induced reversal of cardiac myofibroblasts needs to be explored further.

## 1. Introduction

Cardiac fibrosis is the consequence of production and secretion of excessive extracellular matrix (ECM) proteins, such as: collagens, fibronectin, and elastin, etc., in myocardium. It causes cardiac remodeling and stiffness and deteriorates cardiac functions. Cardiac fibrosis can proceed to cardiac dysfunction and ultimately to chronic heart failure. Cardiac fibrosis is one of the major factors causing high morbidity and mortality in heart disease [1,2,3]. 

Cardiac fibroblasts are critical for keeping heart shape and elasticity as well as normal cardiac function [4]. Under pathophysiological circumstances, myofibroblasts differentiated from fibroblasts secrete collagens and other ECM proteins that limit cardiac functions and underlie the basis of cardiac fibrosis because of myocardial infarction or other non-ischemic risk factors, such as: diabetes, hypertension, or valvular diseases [5]. Transforming growth factor β (TGF-β) is highly expressed in patients with heart failure or dilated cardiomyopathy and plays a critical role in cardiac hypertrophy and dysfunction [6,7,8]. TGF-β is recognized to be the predominant cytokine to trigger differentiation from fibroblasts to myofibroblasts via Smad2/3-dependent or independent pathway [9]. Inhibition of TGF-β receptor activation and subsequent signaling pathway prevents formation of myofibroblast and reduces fibrosis [9]. Cardiac fibrosis usually precedes the occurrence of cardiac functional change and symptoms. Strategies to reverse myofibroblasts to fibroblasts or dedifferentiation of formed myofibroblasts, which is a process to revert to an inactive phenotype characteristic of myofibroblast precursor cells, to resolve fibrosis have emerged recently and have clinical relevance [10,11,12].

Myofibroblasts were traditionally considered to be terminally differentiated cells and were not reversible. Accumulating evidence indicates that myofibroblasts have capacity for dedifferentiation, a process which is defined as the loss of α-SMA, the hallmark for myofibroblasts [9]. Promotion of myofibroblast dedifferentiation may represent a novel mechanism for fibrosis resolution [10,11,12,13,14,15].

Some molecules have been demonstrated to dedifferentiate myofibroblasts. Both prostaglandin E_2_ (PGE_2_) and fibroblast growth factor 2 (FGF2) are molecules that dedifferentiate lung myofibroblasts and corneal myofibroblast, respectively [16,17]. PGE_2_ induces dedifferentiation of human lung myofibroblasts via cAMP/PKA pathway whereas FGF2 utilizes MEK/ERK1/2 pathway to dedifferentiate myofibroblasts [13]. The dedifferentiated myofibroblasts induced by the two molecules have distinct transcriptomic and phenotypic transitions. FGF2 promotes cell proliferation and survival but PGE_2_ suppresses proliferation and survival [13].

Phorbol 12-myristate 13-acetate (PMA) is a potent protein kinase C (PKC) activator that binds to the C1 domain of classical and novel PKC enzyme isoforms [18]. PMA also enhances activity of COX-2 and stimulates production of PGE_2_ in neonatal rat cardiac myocytes via PKC and MAPK activation [19]. PMA induces NK-κB-dependent gene expression in a reporter assay [20].

**Figure 1:**
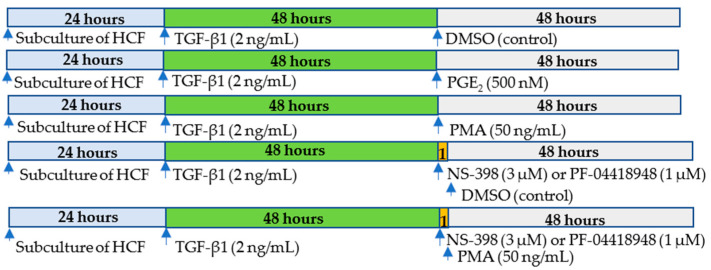
Protocol of studies on dedifferentiation of de novo human cardiac myofibroblasts and activation of COX-2/PGE_2_. TGF-β1 at 2 ng/mL was added into medium for 48 h to differentiate human cardiac fibroblasts to myofibroblasts. After changing medium withoutTGF-β1, cells were treated with PMA, PGE_2_, or DMSO as vehicle control. To investigate the involvement of COX-2/PGE_2_ activation in dedifferentiation of myofibroblasts, NS-398 (a selective COX-2 inhibitor) or PF-04418948 (a selective EP2 receptor antagonist) was added 1 hour before the treatment. The protein expression of α-SMA was detected at the end of 48 hours after treatment of the above agents. HCF: Human cardiac fibroblast.

The effect of COX-2/PGE_2_ pathway on dedifferentiation of human cardiac myofibroblasts remained unknown. In the present study, we investigated this by utilizing de novo myofibroblasts differentiated from human cardiac fibroblasts with TGF-β1 (Figure 1). The expression of α-SMA, a marker for myofibroblasts, was determined by using immunostaining and Western blotting. Our results indicated that TGF-β1 stimulates differentiation of human cardiac fibroblasts to myofibroblast and PMA but not PGE_2_ down-regulates expression of α-SMA from cardiac myofibroblasts, indicating dedifferentiation of de novo human cardiac myofibroblasts. Activation of COX-2/PGE_2_ pathway is not involved in PMA-induced dedifferentiation of myofibroblasts. Both endogenous and exogenous PGE_2_ has no effect on dedifferentiation of human cardiac myofibroblasts.

## 2. Results

### 2.1. PMA but Not PGE_2_ Dedifferentiates De Novo Human Cardiac Myofibroblasts

**Figure 2:**
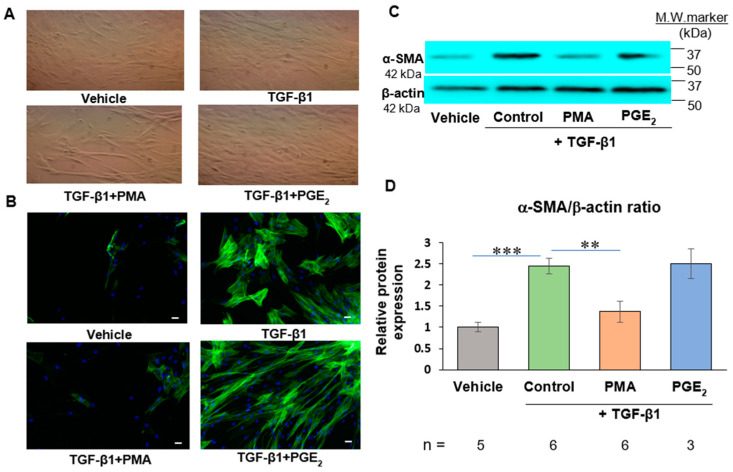
PMA but not PGE_2_ dedifferentiates de novo human cardiac myofibroblasts induced by TGF-β1 from human cardiac fibroblasts (HCFs). (**A**). Phase-contract images of HCFs or human cardiac myofibroblasts treated with PMA or PGE_2_ were shown. Magnification: 100×. (**B**). Double immunostaining of α-SMA (green) and DAPI (blue) in HCFs or human cardiac myofibroblasts was performed. Increased expression of α-SMA was demonstrated in cardiac myofibroblasts after exposure to TGF-β1 at 2 ng/mL for 48 h. PMA at 50 ng/mL reduced expression of α-SMA. PGE_2_ at 500 nM did not affect the increased α-SMA expression in cardiac myofibroblasts. Scale bar = 20 µm. (**C**). Representative images of protein expression of α-SMA in cardiac myofibroblasts were shown. Beta-actin was used as a loading control. (**D**). Immunoblot analysis showed increased protein levels in cardiac myofibroblasts treated with TGF-β1. PMA reduced expression of α-SMA. PGE_2_ did not affect increased α-SMA expression in cardiac myofibroblasts. n: independent samples per group. Bar: mean ± SEM. ** *p* < 0.01, *** *p* < 0.001.

TGF-β1-untreated human cardiac fibroblasts (HCFs) manifested typical morphology of cultured fibroblasts with stellate shape and dendritic extension (Figure 2A). TGF-β1 at 2 ng/mL for 48 h induced changes of cell morphology, which became flattened with irregular shape and larger size than HCFs (Figure 2A). These de novo cardiac myofibroblasts became more three-dimensional shapes after treatment with PMA at 50 ng/mL for 48 h than fibroblasts or myofibroblasts in the control group. PGE_2_ at 500 nM exhibited no effect on the shapes of de novo human cardiac myofibroblasts (Figure 2A).

As shown in Figure 2B, there was barely detectible amount of α-SMA in the vehicle control group indicating low numbers of human cardiac myofibroblasts without stimulation of TGF-β1. After exposure of HCFs to TGF-β1 2 ng/mL for 48 h, the expression of α-SMA was increased indicating differentiation of HCFs to cardiac myofibroblast. Treatment of human cardiac myofibroblasts with PMA at 50 ng/mL for 48 h reduced the expression of α-SMA, signifying the reduction of human cardiac myofibroblasts. Treatment of human cardiac myofibroblast with exogenous PGE_2_ at 500 nM for 48 h had no effects on expression of α-SMA in human cardiac myofibroblasts compared to the TGF-β1 group. Similar results were obtained in protein expression of α-SMA of human cardiac myofibroblasts using Western blotting (Figure 2C,D).

### 2.2. PMA Enhances PGE_2_ Content from Human Cardiac Myofibroblasts

**Figure 3:**
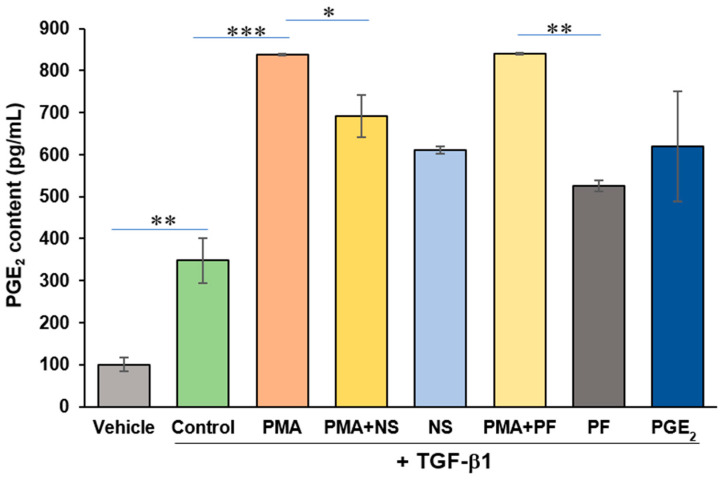
PGE_2_ content in human cardiac myofibroblasts treated with PMA or other indicated agents. Levels of PGE_2_ in the culture medium of cardiac myofibroblasts were measured using PGE_2_ ELISA Monoclonal Kit. Samples and standards were assayed in parallel. All assays were performed in duplicate plates. The results were calculated according to the standard curve of PGE_2_ and expressed as pg/mL. PMA: phorbol 12-myristate 13-acetate; NS: NS-398 (a selective COX-2 inhibitor); PF: PF-04418948 (a selective EP2 receptor antagonist). Data were expressed as mean ± S.E.M. *n* = 4/per group. * *p* < 0.05, ** *p* < 0.01, *** *p* < 0.001.

PGE_2_ is the direct enzymatic product of COX-2. PMA increases activity of COX-2 and production of PGE_2_ from cardiac myocytes [19]. Exogenous PGE_2_ has been shown to stimulate dedifferentiation of fetal and adult lung myofibroblasts [13,15,16]. The content of PGE_2_ released from human cardiac myofibroblasts was determined by using PGE_2_ ELISA kit in the presented studies. As shown in Figure 3, PMA remarkably increased formation of PGE_2_ (*p* < 0.001). NS-398, a selective COX-2 inhibitor, attenuated PMA-induced increase of PGE_2_ (*p* < 0.05). PF-04418948, a potent and selective EP2 receptor antagonist, exerted no effects on increased formation of PGE_2_ induced by PMA. Exogenous addition of PGE_2_ had no effect on the formation of PGE_2_.

### 2.3. PMA-Induced Dedifferentiation of Human Cardiac Myofibroblasts Does Not Depend on Activation of COX-2/PGE_2_ Pathway

**Figure 4:**
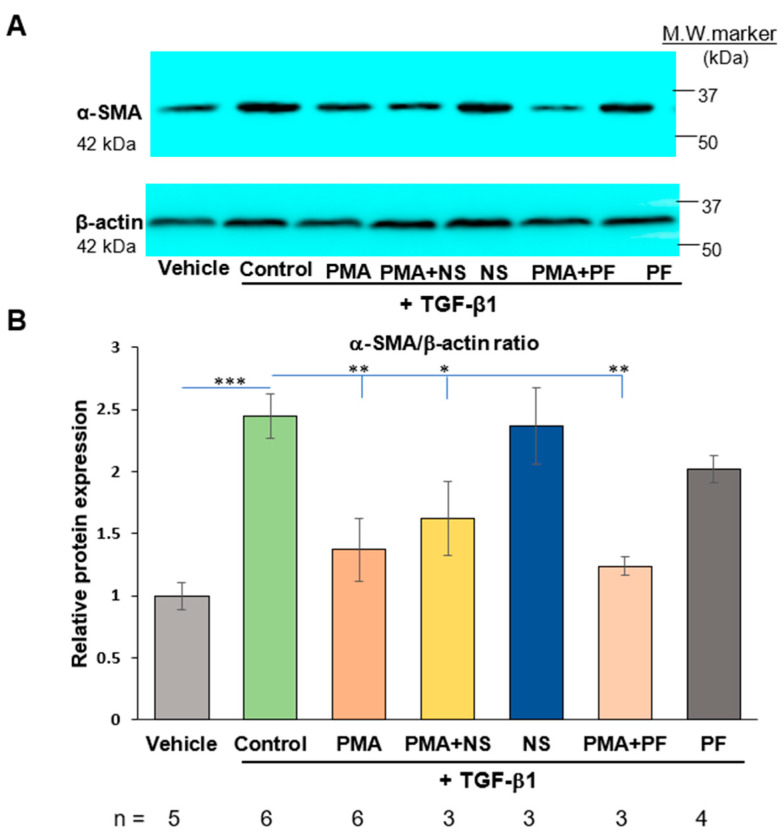
PMA-induced dedifferentiation of cardiac myofibroblasts does not depend on activation of COX-2/PGE_2_ pathway. (**A**). A representative image of Western blotting results of protein expression of α-SMA was shown. Beta-actin (42 kDa) was used as a load control. (**B**). Immunoblot analysis showed a marked increase in α-SMA protein levels in human cardiac myofibroblasts treated with TGF-β1. PMA at 50 ng/mL reduced protein expression of α-SMA. Pretreatment with NS-398 or PF-04418948 had no effect on reduced expression of α-SMA induced by PMA. PMA: phorbol 12-myristate 13-acetate; NS: NS-398; PF: PF-04418948. n: independent samples per group. Bar: mean ± SEM. * *p* < 0.05, ** *p* < 0.01, *** *p* < 0.001.

To investigate the mechanism of PMA-induced dedifferentiation of human cardiac myofibroblasts and association of dedifferentiation on myofibroblasts with activation of COX2/PGE_2_ pathway, NS-398 or PF-04418948 was added into cell medium prior to addition of PMA (Figure 1). As shown in Figure 4, both NS-398 and PF-04418948 had no effects on PMA-reduced expression of α-SMA. These results indicated that activation of COX-2 enzyme and PGE_2_ receptor was not involved in PMA-induced dedifferentiation of human cardiac myofibroblasts.

### 2.4. Activation of COX-2/PGE_2_ Does Not Induce Dedifferentiation of Human Cardiac Myofibroblasts

PGE_2_ increases expression of both EP2 and EP4 receptors. EP2 receptor activation enhances expression of TGF-β1, collagen I and III in H9C2 cardiac myocytes [21]. PGE_2_ is the direct enzymatic product of COX-2. Exogenous PGE_2_ has been shown to dedifferentiate fetal and adult lung myofibroblasts in recent studies [13]. To investigate effects of endogenous PGE_2_ in myofibroblast dedifferentiation, COX-2 inhibitor and antagonist of prostaglandin EP2 receptor were utilized in the presented studies. As shown in Figure 4. TGF-β1 enhanced formation of α-SMA. Both NS-398 and PF-04418948 had no effect on TGF-β1-induced increased expression of α-SMA in human cardiac myofibroblasts. Addition of exogenous PGE_2_ directly to the cell culture medium of cardiac myofibroblasts had no effects on TGF-β1-induced increased expression of α-SMA (Figure 2). These results indicated that both exogenous and endogenous PGE_2_ is not associated with dedifferentiation of human cardiac myofibroblasts.

## 3. Discussion

The major finding of the present study unravels that PMA induces dedifferentiation of de novo human cardiac myofibroblasts and enhances formation of PGE_2_ from these myofibroblasts. PMA-induced dedifferentiation of myofibroblasts is independent of activation of COX-2/PGE_2_ pathway.

During cardiovascular disease or if the heart is subjected to injury such as ischemic insult, cardiac fibroblasts undergo a transition from fibroblasts to myofibroblasts, which is the dominant cell type to generate ECM proteins in cardiac fibrosis. Cardiac fibroblasts are usually associated with the heart with normal functions whereas myofibroblasts are increased in patients with heart failure [11]. Myofibroblasts isolated from patients with cardiomyopathy up-regulate expression of TGF-β1 and profibrotic molecules, such as: collagen I and III. Myofibroblasts TGF-β1 is the essential molecule to promote differentiation of fibroblast into myofibroblasts. Agents to prevent formation of myofibroblasts from fibroblasts have been studied extensively. An effective way to dedifferentiate formed myofibroblasts for fibrosis resolution is a novel approach as cardiac fibrosis with formed myofibroblasts often precedes symptoms of heart disease. Molecules and related mechanism of dedifferentiation of myofibroblasts remain incompletely understood [10]. Molecules targeting TGF-β1 receptor kinase or statins promote dedifferentiation of myofibroblasts isolated from patients with heart failure [11,12].

PMA is a potent activator of PKC, which enhances activation of COX-2/PGE_2_ pathway. PMA has been shown to increase activity of COX-2 and production of PGE_2_ in neonatal rat cardiac myocytes and human pulmonary epithelial cells [19,22]. In the present study, PMA induced dedifferentiation of de novo human cardiac myofibroblasts generated from human cardiac fibroblasts after stimulation of TGF-β1. Both endogenous and exogenous PGE_2_ did not exhibit any effect on dedifferentiation of human cardiac myofibroblasts. PGE_2_ is the direct enzymatic product of COX-2 in varieties of cell types and plays regulatory roles through activation of PGE_2_ receptors. Selective inhibition of COX-2 with NS-398 down-regulates the formation of endogenous PGE_2_ [23]. PF-04418948 is a selective and potent prostaglandin EP2 receptor antagonist that antagonizes the bioactive effects of PGE_2_ [24].

**Figure 5:**
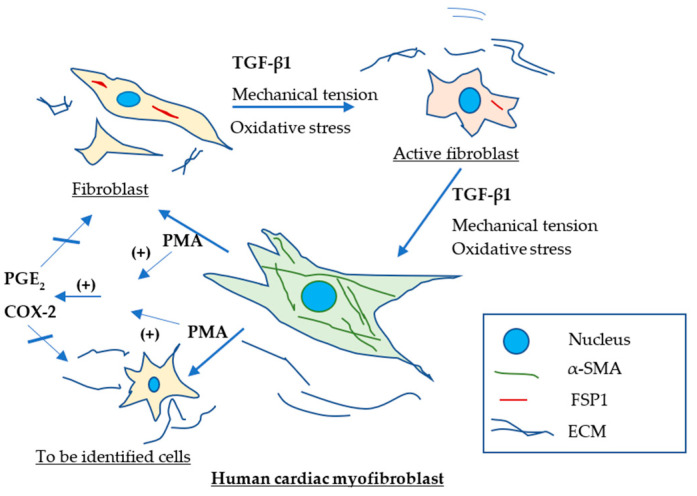
PMA-induced dedifferentiation of human cardiac myofibroblasts does not depend on activation of COX-2/PGE_2_ pathway. A schematic outlines dedifferentiation of cardiac myofibroblasts induced by PMA and the role of COX2/PGE_2_ activation in dedifferentiation of myofibroblasts. TGF-β1 and/or other factors, such as increased mechanical tension, inflammation, or oxidative stress promote differentiation of human cardiac fibroblasts to myofibroblasts though active fibroblasts. PMA dedifferentiates cardiac myofibroblasts to fibroblasts and/or other unidentified cell type. PMA increases activity of COX-2 and formation of PGE_2_. Deactivation of COX-2 and PGE_2_ produces no effects on PMA-induced dedifferentiation of cardiac myofibroblasts. (+) denotes increase.

In the presented study, PMA increased synthesis of PGE_2_ in human cardiac myofibroblasts. COX-2 inhibition attenuated this effect whereas PGE_2_ receptor EP2 antagonist had no effect on PGE_2_ formation. The results suggested that PMA activates COX-2 and increases synthesis of PGE_2_ in cardiac myofibroblasts. Nevertheless, both COX-2 inhibitor and EP2 receptor antagonist had no effects on PMA-induced dedifferentiation of myofibroblast or augmented expression of α-SMA in human cardiac myofibroblasts stimulated by TGF-β1. Direct addition of exogenous PGE_2_ to cell culture medium had no effects on expression of α-SMA from myofibroblasts. These results indicates that both endogenous and exogenous PGE_2_ is not involved in dedifferentiation of de novo human cardiac myofibroblasts (Figure 5).

PGE_2_ has been shown to dedifferentiate lung myofibroblasts [15,16]. Recent studies indicated that cAMP/PKA activation is involved in PGE_2_-induced dedifferentiation [13]. This discrepancy with the presented study on cardiac myofibroblasts may be caused by different organs that fibroblasts reside in or the regulatory role of COX-2/PGE_2_ in different cell lines and organs. It is the general assumption that all fibroblasts bear identical or similar genotypes and phonotypes in the living body. Nevertheless, a study has shown that cardiac fibroblasts are different from fibroblasts residing in the skin and the lungs [25]. Cardiac fibroblasts express significant amount of anti-apoptotic protein Bcl-2, whereas no levels of Bcl-2 were detected in both skin and lung fibroblasts. This discrepancy indicates that those cardiac fibroblasts are more resistant to apoptosis than dermal or pulmonary fibroblasts [25].

The origin and functional heterogeneity of fibroblasts occur. In comparison to vascular mural cells, recent single-cell transcriptomic data reveal extensive inter-organ and intra-organ transcriptional diversity among fibroblasts. The fibroblast diversity primarily reflects differences in ECM production in an organ- and location-specific manner [26]. Intrinsic mechanisms such as transcriptional regulatory networks and epigenetic processes and extrinsic factors, including cell–cell signaling, soluble signaling mediators, or ECM elements [27,28]. Disease states may also cause dynamic changes of fibroblasts [29].

COX-2/PGE_2_ signaling plays a critical role in pulmonary fibrosis. PGE_2_ limits lung fibroblast proliferation, migration, and collagen secretion [30]. Inhibition of COX-2 with NS398 reduces formation of PGE_2_ and improves cardiac contractile force [31]. In contrary to inhibition of lung fibroblast proliferation, PGE_2_ stimulates cardiac fibroblast proliferation and p42/44 MAPK pathway [32]. PGE_2_ up-regulates expression of EP2 and EP4 as well as levels of fibrosis-associated protein expression such as TGF-β1, collagen I and III in H9C2 cardiac myocytes [21]. Selective antagonism of EP2 receptor with AH6809 blocks increased expression of TGF-β1, collagen I and III in both cardiac myocytes and a chronic kidney disease-associated cardiac hypertrophy and fibrosis [20]. PGE_2_ induces cardiac fibrosis by promoting expression of α-SMA and CTGF, fibronectin, and collagen I in cardiac fibroblasts [33].

Although PMA exhibits diverse effects via COX-2-PGE_2_ signaling transduction, the dedifferentiation of cardiac myofibroblasts induced by PMA is COX-2/PGE_2_-independent. The mechanism of PMA-induced dedifferentiation of cardiac myofibroblast needs to be explored. FGF2 has been shown to dedifferentiate cultured corneal myofibroblasts and human lung myofibroblasts [17,34,35]. MEK/ERK mediates FGF2-induced myofibroblast differentiation [13]. It was reported that phorbol ester-sensitive PKC isoforms contribute to the basic FGF-induced coronary smooth muscle cell mitogenesis [36]. The involvement of FGF2 and the associated signaling pathway in PMA-induced cardiac myofibroblast differentiation will be explored in future studies.

Recently, a transcriptional switch has been proposed to govern fibroblast activation in heart disease. Transcriptional factor MEOX1 is especially expressed in activated fibroblasts [37]. Blockage of TGF-β1 signaling reverses the myofibroblast phenotype [11]. In the future, animal model with heart injury and transcriptional profiles of cardiac myofibroblasts will be explored for PMA-induced dedifferentiation of cardiac myofibroblasts. With the exploration of novel mechanism of this action, molecules targeting on myofibroblast dedifferentiation may be developed for antifibrotic therapy.

In conclusion, PMA induces dedifferentiation of de novo myofibroblasts generated from human cardiac fibroblasts upon stimulation of TGF-β1. Activation of COX-2/PGE_2_ pathway is not involved in PMA-induced dedifferentiation of myofibroblasts. Both endogenous and exogenous PGE_2_ has no effect on dedifferentiation of cardiac myofibroblasts.

## 4. Materials and Methods

### 4.1. Antibodies and Regeagents

Primary antibodies: Alpha-SMA (#14968) and anti-β actin (#4970) for Western blotting were purchased from Cell Signaling Technology (Danvers, MA, USA). Anti-α-SMA (#A2547) for immunofluorescence staining was purchased from Sigma-Aldrich (St. Louis, MO, USA). Secondary antibody: Goat anti-rabbit IgG-HRP (#sc-2004) was purchased from Santa Cruz Biotech (Dallas, TX, USA). Goat Anti-Mouse IgG (#31430), SuperSignal^TM^ West PicoPLUS Chemiluminescent Substrate (#34577), and Fluorochrome-conjugated secondary antibody (Alexa Fluor^®^ 488), were purchased from Thermo Fisher (Waltham, MA, USA).

Human cardiac fibroblasts (#6300) and culture medium-Fibroblast Medium-2 (#2331) were purchased from ScienCell Research Laboratories (Carlsbad, CA, USA). All pharmacological agents were reconstituted in DMSO as stock solutions and stored at −20 °C with the exception of TGF-β1 as indicated in the method. PMA (#1585) and DMSO (#8418) were purchased from Sigma-Aldrich (St. Louis, MO, USA). PGE_2_, NS-398 (#70590) and PF-04418948 (#15016) were purchased from Cayman Chemical (Ann Arbor, MI, USA). Recombinant human TGF-β1 protein (#7754-BH) was purchased from R&D Systems, Inc (Minneapolis, MN, USA). Cell lysis buffer (#9803S) was purchased from Cell Signaling Technology (Danvers, MA, USA). BCA Protein Assay Kit-Pierce^TM^ (#23225) was purchased from Thermo Scientific (Waltham, MA, USA). PGE_2_ ELISA Kit-Monoclonal (#514010) was purchased from Cayman Chemical (Ann Arbor, MI, USA).

### 4.2. Cell Culture and Treatment

Human cardiac fibroblasts (HCFs) were cultured and maintained in Fibroblast Medium-2 supplemented with 10% FBS and 100 U/mL penicillin/streptomycin according to manufacturer’s instructions (#6300, ScienCell Research Laboratories, Carlsbad, CA, USA). Trypsin-EDTA solution (0.05%) was used for subculturing fibroblasts. All cell culture and assays in the present study were performed at temperatures of 37 °C, 95% sterile air and 5% CO_2_ in a saturation humidified incubator. Cells were starved with FM-2 without FBS for 12 h then treated with 2 ng/mL of TGF-β1 for 48 h to induce formation of de novo myofibroblasts [16]. NS398 at 3 µM or PF04418948 at 1 µM was added to the cell culture medium 1 h prior to DMSO or PMA [23,24,36,38]. Then, myofibroblasts were treated with DMSO at 0.05% (Vehicle control) or PMA at 50 ng/mL or PGE_2_ at 500 nM for 48 h [16].

### 4.3. Western Blotting

After treatment with indicated agents, lysis of de novo myofibroblasts from HCFs was prepared according to the manufacturer’s instructions (#9803S, Cell Signaling Technology, Danvers, MA, USA). The concentrations of proteins were measured by using Pierce^TM^ BCA Protein Assay Kit. Protein separation and detection were performed according to the previous report [39]. In brief, proteins were loaded and separated with SDS-PAGE. Proteins were electro-transferred to nitrocellular (NC) membrane. After transferring, the NC membrane was incubated with primary antibodies (α-SMA 1:1000, #14968, Cell Signaling Technology, Danvers, MA, USA) at 4 °C overnight. The membranes were incubated with goat anti-rabbit IgG-HRP (1:5000, #sc-2004, Santa Cruz Biotech, Dallas, TX, USA) for 1 h at room temperature. NC membranes were incubated with LumGLO SuperSignal^TM^ West PicoPLUS Chemiluminescent Substrate (#34577 Thermo Fisher, Waltham, MA, USA) for 1 min at room temperature. The membranes were exposed to FluorChem E System—(ProteinSimple, San Jose, CA, USA). NIH ImageJ (1.53e) was used to quantify the density of protein expression.

Since β-actin, the loading control, has the same molecular weight with α-SMA, the NC membranes were stripped in a mild stripping buffer (1.5 g glycine, 0.1 g SDS, 1 mL Tween 20 in 100 mL water with pH 2.2) and followed the protocol provided by abcam website. The membranes were probed with β-actin (#4970, Cell Signaling Technology, Danvers, MA, USA) by using the same Western blotting protocol described above.

### 4.4. Immunofluorescence Co-Staining

Human cardiac fibroblasts were seeded and cultured in single-chamber slides (#154453, Thermo Fisher Scientific, Waltham, MA, USA) followed by overnight serum starvation. Human cardiac fibroblasts were incubated with TGF-β1 (2 ng/mL, #7754BH, R&D Systems, Minneapolis, MN, USA) for 48 h to generate de novo myofibroblasts, which was treated with PMA for 48 h. Cells were washed briefly with PBS and fixed cells with −10 °C methanol for 5 min at −20 °C and air dried immediately. Specimens were incubated with 2.5% normal goat serum in PBS for 20 min to block non-specific binding of IgG, then washed with three changes of PBS 5 min each. Next, they were incubated with primary antibodies (α-SMA at 1:400, #A2547, Sigma-Aldrich, St. Louis, MO, USA) for 60 min at room temperature then washed with three changes of PBS for 5 min each. The slides were incubated with fluorochrome-conjugated secondary antibody (Alexa Fluor^®^ 488, Thermo Fisher, Waltham, MA, USA). Mounting medium containing DAPI (#P36965, Invitrogen, Waltham, MA, USA) was added. It was air dried and sealed with nail polish. Results were examined using Olympus IX83 Inverted Microscope (Olympus Scientific Solutions Americas Corp. Waltham, MA, USA) with appropriate filters. 

### 4.5. Measurement of PGE_2_ Content

After treatment of human cardiac myofibroblasts with PMA, NS-398, PF-04418948, or PGE_2_ for 24 h, the levels of PGE_2_ in culture media of cardiac myofibroblast were measured using the PGE_2_ Monoclonal ELISA Kit (#514010, Cayman Chemical, Ann Arbor, MI, USA) according to the manufacturer’s instructions. The results were calculated according to the PGE_2_ standard curve and expressed as pg/mL.

### 4.6. Statistical Analysis

Experimental data are presented as means ± SEM. One-way ANOVA was used for statistical analysis of data followed by the Tukey’s multiple comparison post hoc tests of group means. Values of *p* < 0.05 were considered statistically significant.

## Data Availability

The data presented in this study are available upon request from the corresponding author.
